# Missed diagnosis of primary intraosseous squamous cell carcinoma - A case report

**DOI:** 10.6026/973206300211271

**Published:** 2025-05-31

**Authors:** Jyoti Biradar, Vishnuchandra Menon, Jaydeep Nilkanthrao Pol, Girish Anandrao Kadkol, Shyam Khant Vipulbhai, Ishwari Milind Khandave

**Affiliations:** 1 Department of Oral and Maxillofacial Surgery, BVDU Dental College and Hospital, Sangli, Maharashtra, India; 2 2Department of Surgical Pathology, Mahatma Gandhi Cancer Hospital, Miraj, Maharashtra, India

**Keywords:** odontogenic cyst, primary intraosseous squamous cell carcinoma, ameloblastoma, osteomyelitis

## Abstract

Odontogenic epithelial remains are the source of the uncommon malignant central jaw tumour known as primary intraosseous squamous cell carcinoma. Although both jaw bones may be affected, the mandible is typically affected. We describe a 40-year-old man who was first misdiagnosed with a periapical infection involving his lower left wisdom tooth. Two years later, the patient arrived at our outpatient department (OPD) with a significant swelling on the left side of the jaw. Advanced imaging and panoramic radiography showed a lesion with total erosion of the left ramus. The mandibular angle biopsy (Fine Needle Aspiration Cytology)) showed acute suppurative inflammation with a large number of giant cells.

## Background:

Squamous cell carcinoma (SCC) that originates from remnants of odontogenic epithelium and does not initially link to the oral mucosa, primary intraosseous squamous cell carcinoma (PIOSCC) is a rare tumour [1]. The World Health Organization (WHO) classified PIOSCC into three categories in 2005: solid-type carcinoma, carcinoma originating from an odontogenic cyst and carcinoma originating from a keratocystic odontogenic tumor [2]. The diagnostic criteria for PIOSCC are currently unclear and making a precise diagnosis of the disease is frequently difficult. According to some research, an SCC that develops inside the jawbone without involving the oral mucosa is one of the histological definitions of PIOSCC [3, 4]. An estimated 12% of all instances of oral cancer are thought to be PIOSCC. Odontogenic cysts, such as dentigerous cysts and keratocystic odontogenic tumours, are the primary cause of PIOSCCs; residual periapical cysts are the rarest source [5]. Oedema, discomfort and sensory abnormalities are often described clinical symptoms of PIOSCC. A primary tumour in another location must be ruled out prior to the diagnosis of PIOSCC [4]. For the diagnosis of PIOSCC; histological findings are frequently not pathognomonic. There is significant diversity in the radiographic characteristics of PIOSCC within and between types [6]. Solid-types are frequently indistinguishable from benign jawbone lesions with well-defined margins in the initial phase; PIOSCC frequently has an osteolytic look with ill-defined irregular edges. All types of larger PIOSCCs can harm the jawbone in different ways [2]. In this article, a case of PIOSCC is presented and the clinical, radiographic and microscopic features of PIOSCC that contributed to a delayed diagnosis are reviewed. This example contributes to the extremely small number of cases of conclusive PIOSCC diagnosis reported in the literature.

## Case report:

In November 2024, a 40-year-old man presented to OPD of Dept of Oral and Maxillofacial Surgery with a history of painful swelling on the left cheek region for 2 months and progressive reduction in mouth opening since 4 months. The patient reported that in April 2022, he had visited another local dental clinic with a chief complaint related to pain in the left posterior mandibular region. His past medical history included controlled type 2 diabetes. The dentist requested a panoramic radiograph and the diagnosis was a periapical infection related to the left third molar and an extraction was performed based on the patient's history. After extraction, the patient reported continuous pain and an antibiotic regime were initiated and pain subsided eventually. A panoramic radiograph was acquired from his first visit and it revealed an ill-defined radiolucent lesion posterior to the left third molar. The clinical examination record did not mention any observation of an intraoral soft tissue mass or ulceration at that time. An extraoral examination revealed a hard swelling on the left cheek region extending from the left ear lobe to the base of the mandible of size 3 x 3 cm. Submandibular and sublingual lymph nodes of the left side were palpable. Pus discharge was noticed extra orally. Intraoral examination revealed reduced mouth opening and interincisal distance of less than 10 mm. Patient also reported of pus discharge intraorally after which his mouth opening improved.

A FNAC was carried out, which revealed acute supportive inflammation with many giant cells. A 3DCT Face was requested and showed complete erosion of the right ramus, with the lesion extending to the body of the mandible. The radiographic signs were highly suggestive of aggressive neoplastic changes and advanced imaging was therefore recommended. Multi-slice computed tomography with contrast revealed a large soft-tissue mass lesion extending from the inferior orbital margin to the lower neck region. An almost complete absence of the left mandibular ramus and temporomandibular joint (TMJ) was observed, as well as erosion of the distal mandibular body. The left maxillary sinus showed erosion of its posterolateral boundary, mild mucosal thickening and a large cyst with a calcific margin and fluid content. The right TMJ was intact and no osteoarthritic changes were noticed. These imaging features suggested a large, osteolytic, neoplastic lesion. Wide local excision under GA was planned based on the CT and biopsy results (FNAC). As part of the patient's planned procedure, a left supra-omohyoid neck dissection was performed based on the frozen section diagnosis. Together with the left mandibular condyle, a 2.8 x 2.5 cm tumour that had pierced 10 mm into the surrounding soft tissue was removed and sent for HP analysis. The operative site was thoroughly irrigated with a 10% betadine solution mixed with regular saline. Next, the Romo Vac drain was placed, layer-by-layer closure was carried out using synthetic absorbable sutures 3-0 Vicryl and synthetic non-absorbable monofilament sutures 2-0 Ethion. Histopathological analysis of H & E-stained slides revealed intra-osseous well differentiated squamous cell carcinoma with many keratin pearls. A focus of tumour necrosis was also seen in certain areas. Patient was given discharge after 5 days and was referred to an onco-radiologist for chemo and radiotherapy. Patent is being followed up regularly with radiographs and clinical pictures.

## Discussion:

The authors of this case report describe an extremely uncommon instance of PIOSCC. It can be challenging to diagnose PIOSCC because it needs to be distinguished from tumour metastases to the jaw, carcinoma originating from the alveolar mucosa epithelium that has penetrated the bone and carcinoma originating from the maxillary sinus epithelial lining. Because of its rarity, little is known about its incidence, prevalence and pathogenesis [7]. About 1% to 2.5% of all odontogenic tumours are PIOSCC, a rare malignant odontogenic tumour [8]. Males are preferred by PIOSCC (2:1 male-to-female ratio) [2]. While it can happen at any age, the fifth decade of life is when it happens most frequently. The mandibular body and posterior mandible are more frequently affected by PIOSCC than the maxilla. The lesion in this instance impacted the left masticator area and the patient was a 40-year-old man. These results are consistent with those of Huang *et al.* [9] who examined 39 solid-type PIOSCCs at Peking University School and Hospital of Stomatology (Beijing, China) between 1985 and 2006. Their results are consistent with those of Thomas and colleagues [10]. The most frequent cause of PIOSCC may be a reactive inflammatory stimulation, whether there is a genetic component that predisposes the condition [10]. Clinically, depending on its location, size and type, PIOSCC is linked to a variety of symptoms. Pain, swelling, sensory abnormalities and common dental conditions are some of these symptoms [11]. The 40-year-old male patient experienced a tingling sensation in the lower lip. In terms of radiological and clinical features, PIOSCCs resemble odontogenic tumours. Early-stage PIOSCC can occasionally mimic common dental conditions such periodontal and periapical disease, which could cause a delayed or incorrect diagnosis [12]. In our instance, the patient's initial dentist misunderstood the lesion as an inflammatory odontogenic disorder when it was still in its early stages. Radiographically, osteolytic bone alterations are typically characterized by uneven, diffuse and poorly defined edges [13]. The lesion displayed comparable characteristics, including erosion at the inferior mandibular boundary. It can be difficult to make a definitive diagnosis of PIOSCC since it is necessary to rule out alveolar carcinomas that have penetrated the bone from the surface, tumours from the maxillary sinus and malignant tumours that have spread to the jaw from other locations [14].

Osteomyelitis, ameloblastoma, odontogenic keratocyst and odontogenic cancer were the differential diagnosis in this instance. Because the radiographic lesion was damaging and had ragged, poorly defined edges, this differential diagnosis were developed. Chondrosarcoma and osteosarcoma were taken into consideration. Chondrosarcomas are uncommon in the jaws and typically develop at the mandibular angle, the alveolar ridge of the premolar-molar area and the anterior alveolar process of the maxilla. But in their early phases, they grow slowly and without pain. Only 7% of all osteosarcomas occur in the jaw bones; instead, they are more commonly found in the long bones. Furthermore, because of their fast growth rate during the early osteolytic stage, osteosarcomas typically have a moth-eaten appearance. The third decade of life is when it occurs most frequently. The radiographic results in the case reported here did not reveal Codman's triangle or a sunray look, which are frequently observed in osteosarcoma patients [7]. PIOSCC is an SCC that arises inside the jawbones and is caused by remnants of the odontogenic epithelium. Primary intraosseous odontogenic cancer (PIOC) was proposed by the WHO in 1972 [11]. Ameloblastic cancer was subsequently added to the WHO classification [12]. Intraosseous mucoepidermoid carcinoma was introduced to the PIOC classification in 1989 after the classification was further updated in 1984 [15]. The WHO coined the name "PIOSCC" in 2005 and divided it into three categories: solid-type carcinoma, carcinoma originating from an odontogenic cyst and carcinoma originating from a keratocystic odontogenic tumour]. Three distinct etiological processes are shown in the PIOSCC detailed subdivisions. PIOSCC can develop as an SCC from the lining of an odontogenic cyst, as an SCC linked to other benign epithelial odontogenic tumours, or as a solid tumour that invades the marrow space and causes osseous resorption [16]. A histological analysis of the current instance showed that a tumour mass was expanding in the bone, but no evidence of direct invasion of the SCC from the squamous epithelium surrounding it was found. The clinical history from the patient's initial visit also corroborated this judgment. The tumour cells exhibited the traits that define SCC. There were no indications of any additional primary SCC or odontogenic cyst or tumour. To sum up, this case emphasizes how crucial it is to interpret radiographs carefully because, as was the case in this instance, a poor interpretation could cause a delay in diagnosis or even a misdiagnosis.

## References

[1] Bodner L (2011). J Oral Pathol Med..

[2] Williams M.D (2017). Head Neck Pathol..

[3] Matsuzaki H (2012). Oral Surg Oral Med Oral Pathol Oral Radiol..

[4] Suei Y (1994). J Oral Maxillofac Surg..

[5] Woolgar J.A (2013). Head Neck..

[6] Sengupta S (2010). J Oral Maxillofac Pathol..

[7] Thakur A (2017). J Oral Maxillofac Pathol..

[8] Tawfik M.A, Zyada M.M (2010). Oral Surg Oral Med Oral Pathol Oral Radiol Endod..

[9] Huang J.W (2009). Arch Pathol Lab Med..

[10] Thomas G (2001). Int J Oral Maxillofac Surg..

[11] Suei Y (2004). Med Hypotheses..

[12] Elzay R.P (1982). Oral Surg Oral Med Oral Pathol..

[13] Reichart R, Philipsen H.P (2004). Odontogenic tumors and allied lesions..

[14] Grisar K (2016). Oncol Lett..

[15] Waldron C.A, Mustoe T.A (1989). Oral Surg Oral Med Oral Pathol..

[16] Abdelkarim A.Z (2019). Imaging Sci Dent..

